# Detection of ctDNA in plasma of patients with clinically localised prostate cancer is associated with rapid disease progression

**DOI:** 10.1186/s13073-020-00770-1

**Published:** 2020-08-17

**Authors:** Edmund Lau, Patrick McCoy, Fairleigh Reeves, Ken Chow, Michael Clarkson, Edmond M. Kwan, Kate Packwood, Helen Northen, Miao He, Zoya Kingsbury, Stefano Mangiola, Michael Kerger, Marc A. Furrer, Helen Crowe, Anthony J. Costello, David J. McBride, Mark T. Ross, Bernard Pope, Christopher M. Hovens, Niall M. Corcoran

**Affiliations:** 1 Department of Surgery, University of Melbourne, 5th Floor Clinical Sciences Building, Royal Melbourne Hospital, Grattan Street, Parkville, VIC 3050 Australia; 2 Melbourne Bioinformatics, The University of Melbourne, Carlton, VIC 3053 Australia; 3 Department of Medicine, School of Clinical Sciences, Monash University, Melbourne, VIC 3800 Australia; 4 Department of Medical Oncology, Monash Health, Melbourne, VIC 3168 Australia; 5 Illumina Cambridge Ltd., Great Abington, Cambridge, UK; 6 Division of Bioinformatics, Walter and Eliza Hall Institute, Parkville, VIC 3052 Australia; 7 Australian Prostate Cancer Centre, North Melbourne, VIC 3195 Australia; 8 Department of Urology, Royal Melbourne Hospital, Melbourne, VIC 3050 Australia; 9 Department of Urology, Inselspital, Bern University Hospital, Bern, Switzerland; 10 Department of Clinical Pathology, The University of Melbourne, Victorian Comprehensive Cancer Centre, Melbourne, VIC 3000 Australia; 11 Department of Urology, Peninsula Health, Frankston, VIC 3199 Australia; 12 Victorian Comprehensive Cancer Centre, Melbourne, VIC 3000 Australia

## Abstract

**Background:**

DNA originating from degenerate tumour cells can be detected in the circulation in many tumour types, where it can be used as a marker of disease burden as well as to monitor treatment response. Although circulating tumour DNA (ctDNA) measurement has prognostic/predictive value in metastatic prostate cancer, its utility in localised disease is unknown.

**Methods:**

We performed whole-genome sequencing of tumour-normal pairs in eight patients with clinically localised disease undergoing prostatectomy, identifying high confidence genomic aberrations. A bespoke DNA capture and amplification panel against the highest prevalence, highest confidence aberrations for each individual was designed and used to interrogate ctDNA isolated from plasma prospectively obtained pre- and post- (24 h and 6 weeks) surgery. In a separate cohort (*n* = 189), we identified the presence of ctDNA *TP53* mutations in preoperative plasma in a retrospective cohort and determined its association with biochemical- and metastasis-free survival.

**Results:**

Tumour variants in ctDNA were positively identified pre-treatment in two of eight patients, which in both cases remained detectable postoperatively. Patients with tumour variants in ctDNA had extremely rapid disease recurrence and progression compared to those where variants could not be detected. In terms of aberrations targeted, single nucleotide and structural variants outperformed indels and copy number aberrations. Detection of ctDNA *TP53* mutations was associated with a significantly shorter metastasis-free survival (6.2 vs. 9.5 years (HR 2.4; 95% CIs 1.2–4.8, *p* = 0.014).

**Conclusions:**

CtDNA is uncommonly detected in localised prostate cancer, but its presence portends more rapidly progressive disease.

## Background

The majority of men diagnosed with localised prostate cancer are effectively cured with surgery and/or radiation therapy [1]; however, a proportion of patients will experience disease recurrence, often associated with high grade and/or locally advanced tumours [2]. Predicting patients at highest risk of relapse remains challenging, as while clinical prognostic factors are certainly helpful [3], they do not always provide the full picture for an individual patient. The ability to accurately identify these patients may have clinical implications, as they have the potential to benefit from systemic treatment intensification strategies. While the value of these systemic treatments in metastatic hormone-sensitive prostate cancer (mHSPC) is undisputed (ENZAMET [4], ARCHES [5], TITAN [6], STAMPEDE [7, 8], LATITUDE [9], CHAARTED [10]), utility in early-stage disease is less clear. However, this is an area of intense investigation in which treatment strategies will continue to evolve [11, 12]. Prognostic biomarkers to predict risk of metastatic disease development are thus urgently required, not only to facilitate discussions with patients/caregivers around expected outcomes, but also to inform clinical trial design and perhaps ultimately guide optimal management.

A number of molecular biomarkers for use on primary prostate cancer tissue are already in clinical use (for example Decipher [13], Oncotype [14], and Prolaris [15]) and can be used to predict the presence of adverse pathology or recurrent disease post primary treatment. These transcription-based assays require the use of tumour tissue from the diagnostic biopsy specimen, which in the context of the well-described molecular heterogeneity of primary prostate cancer [16] as well as the sampling error inherent with traditional biopsy strategies [17], has led to the exploration of circulating biomarkers as a ‘whole tumour’ sampling technique. An array of different biomarkers have been investigated, including circulating tumour cells (CTCs), exosomes and circulating tumour DNA (ctDNA), although most of these have been in the metastatic castration-resistant setting (mCRPC), where tumour burden is higher [18, 19]. Use of these biomarkers in the localised setting, where disease volume is considerably lower, may pose significant challenges. In this context, the use of ctDNA has some appeal, due to the ability to amplify the signal against background noise, as well as the increasing annotation of particular genomic features as important drivers of clinical outcome [20]. However, the current analytical sensitivity and limit of detection of most ‘off the shelf’ ctDNA assays is inadequate to reliably call low-frequency variants observed in early prostate cancer [21]. One of the ways to overcome this is to use ultradeep sequencing using targeted panels looking for patient-specific alleles.

This personalised assay approach has recently been investigated in localised prostate cancer [22]. As part of a broader attempt to identify ctDNA in a large cohort of patients with localised disease, Hennigan and colleagues performed multiregional sequencing to identify patient-specific variants present clonally or at high allele frequency within the primaries of nine patients, followed by targeted deep sequencing of these variant sites within plasma-derived cell-free DNA. Despite readily detecting ctDNA in patients with metastatic disease, they were unable to positively identify ctDNA in any patient by this or any other approach. Although the authors provide a number of plausible biological reasons for this negative finding, including low rates of cell proliferation and ctDNA shedding in localised disease, as well as more restricted access to the vasculature compared to metastatic lesions, a further possibility is that the number of patients with truly high-risk disease within their cohort was too low to detect a positive signal. Certainly ctDNA measurement has proven feasible and clinically meaningful in detecting the presence of ‘micrometastatic disease’, and be prognostic for recurrence, in other tumour types in the localised setting [23, 24].

In this study, we performed panel-based targeted sequencing informed by individual patient tumour WGS, as well as tagged-amplicon deep sequencing (TAm-Seq) across the *TP53* gene in two separate cohorts of patients with localised prostate cancer, with an emphasis on high-risk disease [25]. We found that ctDNA was detectable in both the pre- and post-operative setting, and that pre-operative detection was associated with a significant reduction in metastasis-free survival.

## Methods

### Study cohorts

Two separate patient cohorts were used for this study. For tumour-based WGS analysis (WGS cohort), we prospectively enrolled 10 consecutive patients presenting to a single surgeon with clinically localised prostate cancer suitable for prostatectomy with curative intent. For the TAm-Seq analysis (TAm-Seq cohort, *n* = 189), patients were selected from a prospectively collected and clinically annotated institutional bio-repository [26]. Patients were prioritised based on the length of follow-up, enriched for high-risk disease and recurrence events. Collection and use of material had Institutional Review Board approval (HREC# 626-14).

### Whole-genome sequencing and analysis

A summary of the blood and prostatectomy specimens available for WGS is shown in Additional file 1: Table S1. Patients with both tumour and matched germline DNA samples from a peripheral blood buffy coat specimen were suitable for WGS to discover patient-specific variants. Genomic DNA extracted from blood and tissue samples were prepared using the TruSeq® DNA PCR-free sample preparation kit (Illumina, San Diego, CA, USA). The libraries were sequenced as paired-ends (2 × 150 cycles) using a HiSeqX platform to an average depth of >30x for germline and >100x for tumour samples, following alignment to the Human Reference sequence (hg19) using iSAAC-03.16.02.19 and removal of duplicate read-pairs. Variant calling was performed using the Illumina IsaacVariantCaller (2.6.53.23) [27].

### Enrichment panel design and targeted sequencing and analysis

A subset of somatic variants identified through whole-genome sequencing were included on the panel for each patient. Variants were preferentially selected for inclusion where they occurred in genes previously implicated in cancer [28] or prostate cancer specifically [21]. A range of SNVs, indels and structural variants (SVs) were targeted. The final design targeted 88 kb of the genome. Target regions were uploaded to Illumina DesignStudio (www.designstudio.illumina.com) for probe design. A single probe was selected for small variants, while SVs were targeted using a probe from each side of the rearrangement junction. For indels, a probe adjacent to the variant was selected (Additional file 2: Figure S1). Probes were validated on fresh frozen tumour DNA to confirm successful design and synthesis, and a list of genomic variants targeted in each patient is provided in Additional file 3: Table S2.

For each patient, blood was collected from up to three timepoints in EDTA tubes for ctDNA analysis; sample (a) was taken pre-surgery, sample (b) 24 h post-surgery and sample (c) 6 weeks post-surgery. Immediately after collection, plasma was collected following a double spin (820*g* for 10 min; 16,000*g* for 10 min) and stored at − 80 °C until analysis. Circulating DNA was extracted using the QIAamp Circulating Nucleic Acid Kit (Qiagen, Hilden, Germany), and DNA quality was assessed using an Agilent BioAnalyzer and quantified using the Qubit method (Thermofisher, Delaware, USA).

Input into library preparations was between 28 and 60 ng of ctDNA, with a median value of 39 ng as determined by Qubit. Enriched libraries were prepared from ctDNA extracted from plasma using the TruSight® Tumor 170 library prep kit supplemented with the TruSight® Oncology UMI Reagents (Illumina Inc.). Adapters containing UMIs enable detection of genuine, very low-frequency variants through the reduction of background noise in sequencing data. Due to the small fragment length of the starting material, the DNA shearing step of the TST170 protocol was omitted. Libraries were enriched with the custom panel then sequenced as paired-ends (2 × 150 bp) plus indexing reads on NextSeq High output. Five or six samples were loaded per flow cell to achieve ~ 40,000x raw depth per sample.

Samples were processed using an Illumina R&D analysis pipeline to take advantage of the UMIs incorporated during library preparation. Reads were grouped into read families by alignment to the hg19 human reference sequence followed by collapsing and stitching into unique fragments utilising start and stop positions, UMI barcodes and the paired read (where present) to generate collapsed BAM files. These collapsed families of reads are low noise and high confidence. Positions targeted by the panel were reviewed using Integrative Genomics Viewer (IGV; The Broad Institute) to confirm the presence or absence of the targeted variants.

### TP53 TAm-Seq

DNA was isolated from archived plasma samples stored in liquid nitrogen using the QIAamp Circulating Nucleic Acid Kit and quantified using Qubit Molecular Probes dsDNA high sensitivity assay kit (Invitrogen, CA, USA). For amplicon production, forward and reverse primers (Additional file 2: Figure S2) tiling across the *TP53* gene were combined for use in eight separate PCR reactions per patient. Input was 2 ng of DNA, which was added to a PCR reaction mix composed of 2 μl of primer (0.5 μl ea. @ 10 μM) and 10 μl Q5 DNA pol 2X master mix made up to 20 μl with MQ water. The reaction was run on a ViiA 7 PCR machine with the following settings: initial denaturation at 95 °C × 1 min, then cycle denaturation at 95 °C × 10s following by annealing at 58 °C × 1 s and then extension at 72 °C × 18 s for 38 cycles. Amplicons were then combined into two pools of non-overlapping products and cleaned with the Qiagen QIAquick PCR purification kit following the manufacturer’s instructions. DNA was quantified using a nanodrop and 100 ng of DNA of each pool end repaired and adaptor ligated using NEBNext Adaptors for Illumina according to the manufacturer’s instructions. Following a AMPure XP bead clean-up, a second round of PCR amplification was performed in a total reaction volume of 50 μl consisting of 20 μl of adaptor ligated DNA fragments, 25 μl Q5 2X master mix, 2.5 μl index primer (10 μM) and 2.5 μl universal PCR primer (10 μM). PCR amplification was then performed in a thermocycler under the following conditions: initial denaturation at 98 °C × 30 s, then cycle denaturation at 98 °C × 10 s following by annealing/extension at 65 °C × 75 s for 8 cycles, with a final extension at 65 °C for 5 min. The reaction mixture was then cleaned again using AMPure XP beads following the manufacturer’s instructions at a ratio of 0.9:1 beads to ligation mixture. The pool was then quantified and quality checked on a Bioanalyzer (Agilent, CA, USA) before sequencing on a MiSeq (Illumina Inc.). A detailed description of the experimental procedure is provided as supplementary information (Additional file 4; Supplementary Methods).

### TAm-Seq analysis

For each sample in the study, paired-end DNA sequencing reads (length 151 bp) were aligned to the human genome reference hg19 using BWA mem (version 0.7.17). Reads were assigned to their corresponding PCR amplicons based on their alignment coordinates. To be considered for further analysis, reads were required to have a minimum alignment length of 75 bp, a minimum overlap of at least 75% of their target amplicon and no more than 2 bp edit distance from the reference genome. Using the filtered set of reads, the frequency of each DNA base was counted at each genomic locus within the target PCR amplicon regions for each sample. Using the control samples, at each locus, a null model was computed to represent the expected distribution of allele counts when no variant is present. Allele count data was modelled (with add-one smoothing) using the Dirichlet Multinomial distribution with best hyperparameter computed by maximising the log-likelihood function using the Newton-Raphson method. For each locus in the case samples, the allele count was compared with the null model. The weight of evidence against the null was measured by the Bayes factor B. The prior distribution of the count data of the case samples was given by a Dirichlet distribution on a 3-simplex, with all pseudocounts set to ½ (as per Jeffrey’s Prior on a multinomial distribution). Variant allele frequencies (VAF) were computed using the maximum a posteriori (MAP) estimate on the posterior distribution marginalised to the component representing the non-reference allele. After filtering out any variants with gnomAD (gnomad.broadinstitute.org) population frequency greater than 1%, a set of high-confidence variants was selected based on having a minimum filtered depth of coverage of 500 reads, a minimum log Bayes factor of 6 (equivalent to posterior odds against the null hypothesis of 50:1) and a cohort frequency of less than four samples. The predicted pathogenicity of detected variants was assessed using Varsome [29].

### Clinical outcomes

Postoperative PSA data was collected prospectively in all TAm-Seq patients. Biochemical recurrence was defined as a single post-operative PSA reading of > 0.2 ng/ml, or a rising measurement below this that was determined to represent a recurrence by the treating physician and led to the institution of salvage therapy. The presence of metastasis and mode of diagnosis was determined by retrospective chart review. For patients without recurrence or metastasis, follow-up was censored at the time of their last PSA or clinical review as appropriate. To compare biochemical recurrence-free and metastasis-free survival between groups, Kaplan-Meier curves were generated and compared using a two-sided logrank test, with significance assumed at *p* < 0.05.

## Results

Paired tumour and germline WGS was completed on eight patients, the clinical and pathological characteristics of whom are shown in Table 1. Consistent with contemporary practice, all patients had intermediate- or high-risk disease at the time of radical treatment, and two patients underwent salvage prostatectomy for radiorecurrent disease. Patients at risk of metastasis based on their disease characteristics were staged pre-operatively with a technetium-99 m-MDP whole body bone scan and cross-sectional imaging (MRI pelvis + lumbar spine or CT abdomen/pelvis) as appropriate. No patient had evidence of metastatic disease at the time of surgery.

**Table 1:** Clinical and pathological characteristics of the WGS study cohort

Sample	Age	PSA	Prior RT	cT	Bx GG	Staging	RP GG	pT	Vol (cc)
11193_2	70	26.9	y	cT1c	5	MRI WBBS	5	pT3b	21.3
11196_3	57	9.3	y	cT1c	3	MRI WBBS	3	pT3b	2.1
11201_4	64	8.1	n	cT3a	5	CT WBBS	5	pT3b	18.5
11199_5	67	7.8	n	cT2a	2	MRI	3	pT3a	15.2
11219_6	58	6.5	n	cT1c	2	MRI	2	pT3a	2.1
11231_9	59	6.1	n	cT1c	2	MRI	2	pT2c	3.7
11242_10	61	4.1	n	cT2a	2	CT WBBS	2	pT3a	3.7
11243_11	76	7.9	n	cT2a	5	CT WBBS	3	pT3a	7.5

*PSA* prostate-specific antigen, *RT* radiotherapy, *cT* clinical stage, *Bx* diagnostic biopsy, *GG* International Society of Urological Pathology Grade Group, *RP* radical prostatectomy, *pT* pathological stage, *Vol (cc)* tumour volume (cm^3^)

**Fig. 1:**
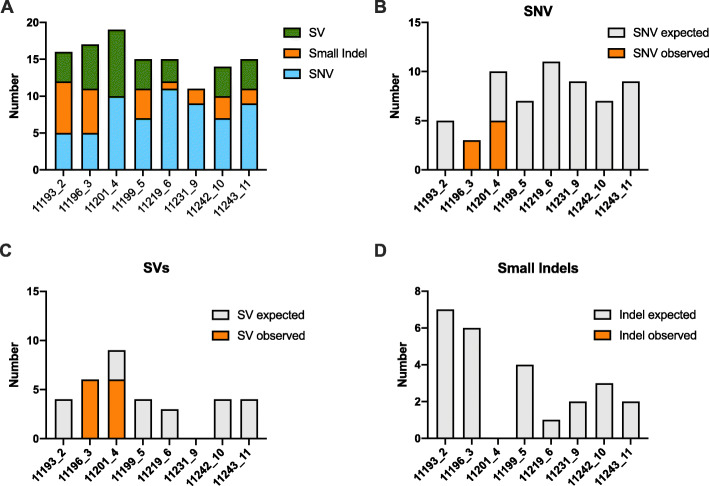
**a** Targeted genomic variants in each patient by category. Per patient summary of **b** SNVs, **c** SVs and **d** small indels targeted and detected in any ctDNA sample by deep targeted sequencing

The number of genomic aberrations identified by tumour WGS per patient is summarised in Additional file 1: Table S3. The average mutation rate was 5.51 SNVs per megabase, consistent with a high-risk disease cohort [21]. The category of features selected for targeted capture and sequencing and a breakdown of variants targeted versus those detected in ctDNA are summarised in Fig. 1. Genomic features of prostate cancer were identified in ctDNA in two of eight patients investigated with only SNVs and SVs being detected. Interestingly, while all SNVs and SVs targeted in patient 11196_3 were detectable in the ctDNA, we could find no evidence of the targeted small indels, despite their presence at high frequency in the tumour WGS data and good coverage in these regions. Similarly CNAs detected in ctDNA in general correlated poorly with tumour WGS and could not be reliably called at the ctDNA levels observed in this localised cohort.

**Fig. 2:**
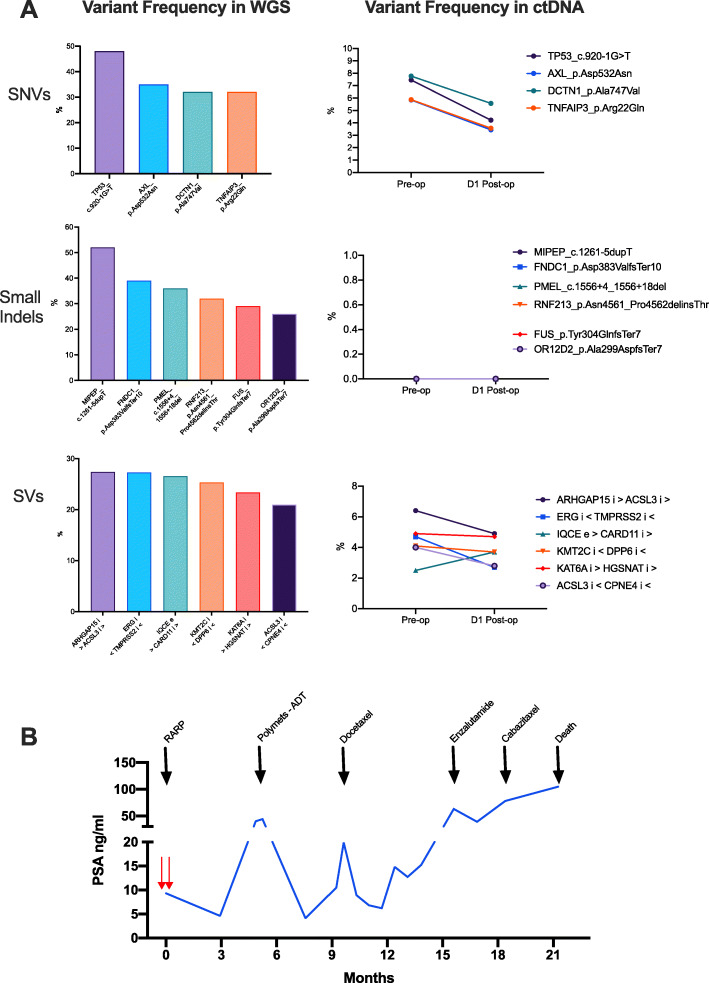
**a** Selected variant frequency observed in tumour whole-genome sequencing (WGS) and circulating tumour DNA (ctDNA) at indicated timepoints in patient 3. **b** Summary of clinical history of patient 3 post-surgery with ctDNA sampling indicated by red arrows

**Fig. 3:**
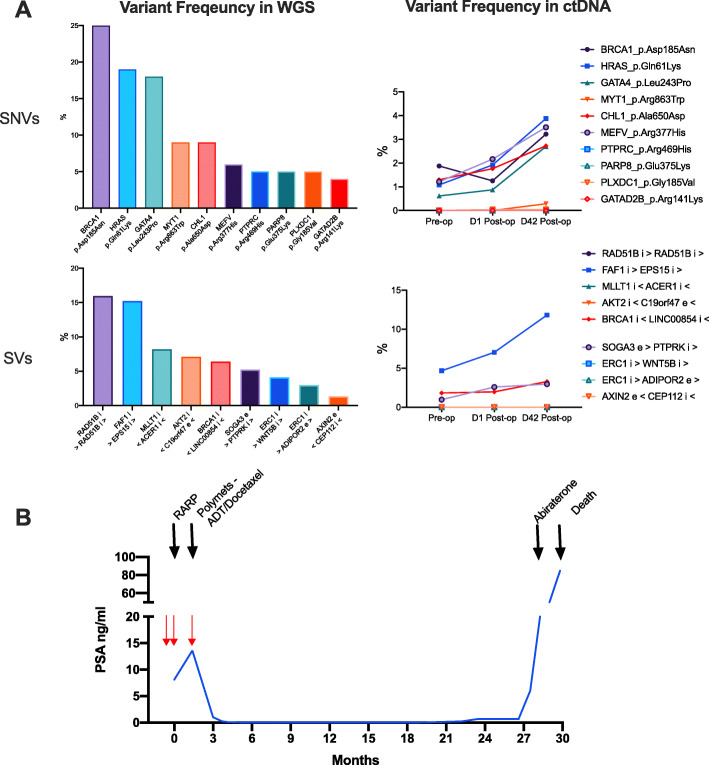
**a** Selected variant frequency observed in tumour whole-genome sequencing (WGS) and circulating tumour DNA (ctDNA) at indicated timepoints in patient 4. **b** Summary of clinical history of patient 4 post-surgery with ctDNA sampling indicated by red arrows

Changes in specific variants identified in the ctDNA in patients 11196_3 and 11201_4 over time are shown in Figs. 2 and 3, along with a summary of the clinical course of their disease post-surgery. Two timepoints (pre-operative and 24 h post-surgery) were available for patient 11196_3, who showed some evidence of a treatment effect, with a decrease in the variant allele frequency observed for almost all of the ctDNA variants measured (Fig. 2a). A similar effect was not consistently observed in patient 11201_4, with an inexorable increase in variant allele frequency of measured variants observed across the three timepoints. Although all patients were negative for metastatic disease using conventional imaging at the time of surgery, patients in whom ctDNA was detectable pre-treatment had strikingly aggressive post-treatment clinical courses compared to patients with no detectable ctDNA (Additional file 2: Figure S3), characterised by early disease recurrence and metastases, relatively poor response to systemic treatments, and progression to death within 2.5 years.

**Fig. 4:**
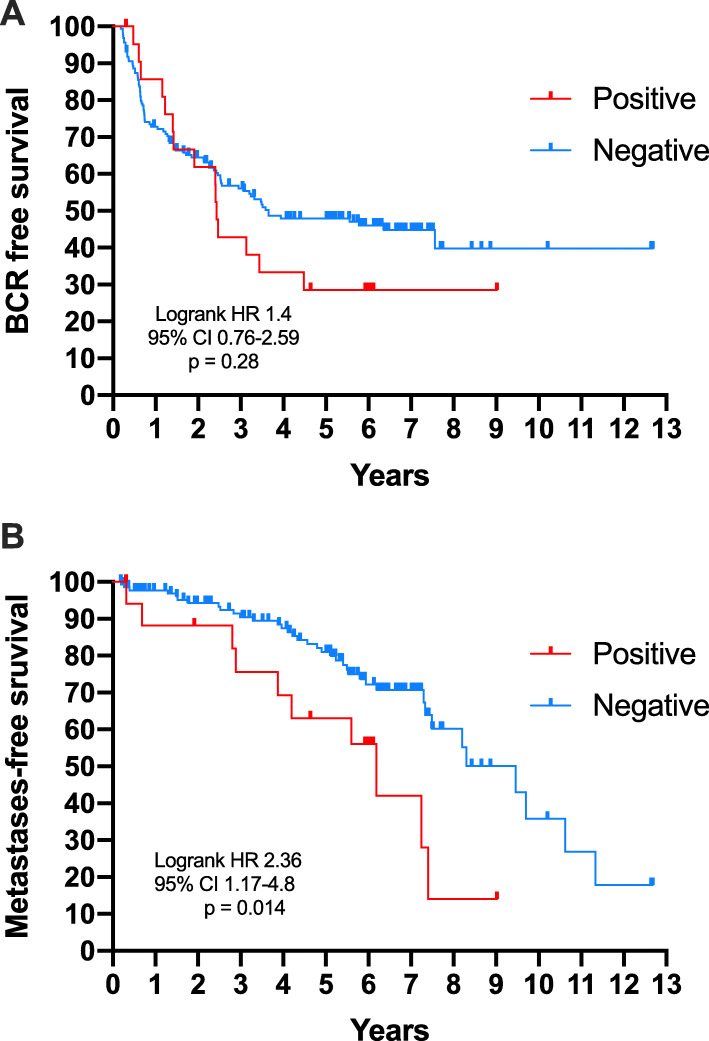
Kaplan-Meier curves demonstrating **a** biochemical recurrence and **b** metastasis-free survival in a cohort of 189 patients undergoing prostatectomy categorised by pre-treatment *TP53* ctDNA status

To extend these findings, we performed targeted sequencing of plasma DNA collected from patients undergoing prostatectomy for presumed localised prostate cancer (*n* = 189), focussing on *TP53*, one of the most commonly mutated genes in prostate cancer, and one that is positively enriched for in metastatic disease (including in patient 11196_3) [20, 30]. As indicated in the study description above, the cohort was selected to enrich for patients with high-risk and recurrent disease, to maximise the chances of detecting ctDNA in some patients, the clinical and pathological characteristics of whom are summarised in Table 2. After filtering, the mean depth of coverage within the target regions was 3198 reads. Positive detection of *TP53* ctDNA variants was identified in 22 cases (12%), with no variants detected in the remainder (Additional file 1: Table S4). The median and mean coverage depth for called variants was 9892x and 12,695x respectively. Fourteen out of 21 variants identified were predicted to be pathogenic or likely pathogenic, with only one variant classed as likely benign. There was no significant difference in biochemical recurrence-free survival (BFS) between patients with or without a detectable variant (Fig. 4a), with a mean BFS of 2.4 vs. 3.7 years (HR 1.4; 95% CIs 0.76–2.6, *p* = 0.28). In contrast, metastasis-free survival (MFS) was significantly shorter in patients positive for a variant (Fig. 4b), with mean MFS of 6.2 vs. 9.5 years (HR 2.4; 95% CIs 1.2–4.8, *p* = 0.014). Similar results were observed using a contingency analysis (Additional file 1: Table S5).

**Table 2:** Clinical and pathological characteristics of the TAm-Seq study cohort

*n*	189
Age (years)	
Median	63
IQR	58–67.6
PSA ng/ml	
Median	7.5
IQR	5.3–12.95
cT	
1	105 (55.6%)
2	63 (33.3%)
3	21 (11.1%)
Biopsy ISUP Grade Group	
1	33 (17.5%)
2	61 (32.3%)
3	29 (15.3%)
4	35 (18.5%)
5	31 (16.4%)
pT	
2	88 (46.6%)
3a	60 (31.7%)
3b	41 (21.7%)
Prostatectomy ISUP Grade Group	
1	10 (5.3%)
2	63 (33.3%)
3	61 (32.3%)
4	11 (5.8%)
5	44 (23.3%)
Tumour volume (cc)	
Median	3.35
IQR	1.5–7.37
Biochemical recurrence	
No	90 (47.6%)
Yes	99 (52.4%)
Metastases	
No	117 (61.9%)
Yes	49 (35.9%)
Not reported	23 (12.2%)

## Discussion

Although ctDNA is reported to be relatively abundant in metastatic prostate cancer, at the time of initiation of this study, it was unclear if ctDNA could be detected in patients with localised disease, as well as the clinical implications of its presence. A number of early studies identified an association between total cell-free plasma DNA concentration and the presence of cancer and biochemical recurrence post prostatectomy [31–33]; however, the first study to investigate circulating tumour DNA in this setting has only recently been reported [22]. In this study, Hennigan and colleagues used ultra-low pass whole-genome sequencing of cell-free plasma DNA in over 100 patients with clinically localised disease prior to prostatectomy, as well as deep targeted sequencing of a bespoke panel of single nucleotide variants/indels selected as clonal or major subclonal events, based on individual multiregional whole exome sequencing in a subset of nine patients. Despite the technical robustness of the methodology evidenced by consistent identification of ctDNA in patients with established metastatic disease using these approaches, they were unable to identify any ctDNA in any patient with clinically localised disease.

Here we have been able to identify ctDNA in two of eight patients with localised disease using a personalised approach based on individual whole-genome sequencing followed by deep-targeted sequencing of selected variants in plasma cell-free DNA. Furthermore, in a retrospective analysis of patient samples (enriched for high-risk patients) using the previously described TAm-Seq method to tile across a single gene, we have been able to detect ctDNA in 22 out of a further 189 patients. Importantly, we were able to demonstrate that detection of ctDNA in the pre-prostatectomy setting has prognostic implications and is associated with more rapid progression to metastatic disease in both analyses. This is of clear clinical relevance, given that MFS is a strong surrogate of overall survival in localised prostate cancer [34].

Despite differences in sequencing and analysis methods between the studies, at least for the individual targeted analysis, it is likely that clinical differences between the cohorts are the basis of the observed discrepancy. For instance in the Hennigan study, although 8/9 patients who underwent tissue and plasma cell-free DNA sequencing had locally advanced and/or high grade tumours, only one patient experienced a biochemical recurrence with 24 months of surgery, and no patients were reported to progress to metastasis. In contrast, from our cohort, three patients had primary PSA persistence, and a further two patients experienced biochemical recurrence within 24 months. Of these, ctDNA was only detectable in 2 patients, both of whom had primary PSA persistence and very rapid disease trajectories, characterised by early progression to overt metastatic disease and death. Patient 11196_3 had a moderately sized tumour, with only ISUP grade group 3 disease at prostatectomy, but was radiorecurrent, with extensive extracapsular extension and invasion into the seminal vesicle. Prominent lymphovascular invasion, a pathological feature associated with aggressive disease [35], was also observed in large extraprostatic vessels. The second patient 11201_4 had more conventional high-risk disease, with a large volume, locally advanced ISUP grade group 5 tumour. In both cases, pre-treatment staging with conventional imaging was negative for metastatic disease, although the likelihood of tumour cell dissemination at the time of diagnosis was high. Similarly, the cohort we used for the TAm-Seq analysis is a higher risk cohort than that used for ultra-low pass whole-genome analysis, based on a higher median PSA and the proportion of patients with ISUP grade group 4/5 disease, which is not unexpected given this study group was enriched for recurrent and metastatic events post-prostatectomy. However, there may be some additional technical issues, as despite including probes for copy number variants in the targeted panel for 7/8 patients (including patients 11196_3 and 11201_4), we were unable to positively identify any ctDNA CNAs concordant with tumour whole-genome sequencing data using this particular custom panel. It may be that even in patients with detectable ctDNA in the clinically localised setting, absolute levels are too low to make confident calls with the sensitivity of the assay used in this study, at least not without a considerable investment is assay development and control samples for normalisation. Certainly, it has been suggested that tumour DNA fractions > 35% are required to accurately detect CNA events [36], particularly deletions, and this far exceeds the levels observed in our study, even with extremely aggressive localised disease.

A key finding in this study is the consistent association between pre-treatment ctDNA detection and clinical outcomes, which may serve as a useful adjunct to select patients for treatment intensification at the time of diagnosis. Creating a custom panel based on sequencing of the primary tumour and identifying individual-specific variants may be the most sensitive way of positively identifying ctDNA in patients, but outside of particularly invested centres, its use as a clinical strategy is limited by the cost and turn-around time. An alternative approach is to target common variants identified in a landscape-type analysis [21]. We were able to measure SVs and SNVs consistently, suggesting that a strategy built around detecting this class of variants based on observed frequency in landscape analysis may be the best approach. One concern however is that SNVs in prostate cancer are rarely located in ‘hot-spot’ regions, and breakpoint sites vary significantly between patients, requiring coverage of a relatively large genomic region to increase sensitivity. Certainly using this approach tiling across *TP53*, mutations of which are enriched in patients with metastatic disease, we were able to identify ctDNA in just under 12% of patients, where it again was associated with the development of metastatic disease. Clearly, all patients with potentially detectable ctDNA levels were not identified, as many patients who were *TP53* ctDNA negative also experienced metastatic progression post-operatively. Extending the number of variants assessed will likely increase test sensitivity [37], but for an ‘off-the-shelf’ assay, an optimal ‘sweet spot’ that balances test complexity, cost and diagnostic performance will need to be determined.

There are a number of limitations to our study that warrant some consideration. Undiagnosed clonal haematopoiesis of indeterminate potential (CHIP), of which *TP53* mutation is a relatively frequent driver, has been shown to be a common source of variants detected in deep cell-free DNA analyses and ideally should be controlled for by concomitant sequencing of white blood cell DNA which we have not performed [38, 39]. However, we observe that two of the variants (chr17:7578475_G>A and chr17:7577121_G>A) identified by TAm-Seq were also detected in previous whole-genome sequencing on the same tumour samples [30]. Enrichment for a known driver of prostate cancer spread in the cohort of patients who progress to clinical metastases suggests that the majority of identified variants are tumour related, but further work is required to confirm this. In addition, the TAm-seq assay had reasonably low sensitivity, which was not unexpected given the reasonably low frequency of *TP53* mutations in localised prostate cancer [20, 21]. However despite these issues with sensitivity and specificity, the TAm-Seq analysis supported the association between ctDNA detection and aggressive disease in the localised setting. It is also important to note that all patients in our study were staged using conventional imaging. Given the reported greater sensitivity of molecular staging, for example, PSMA-PET in detecting low volume disease in the recurrence setting [40], such imaging modalities may better detect patients with micrometastatic disease pre-treatment. Whether such imaging gives potentially the same information or is complementary with ctDNA detection will need to be determined.

## Conclusions

In summary, using two separate cohorts and two different approaches, we have found that ctDNA can be identified in patients with clinically localised prostate cancer and is associated with a significantly shorter time to metastatic progression. Efforts to improve levels of detection, specificity and universal applicability of ctDNA detection in this setting are warranted.

## Supplementary information

**Additional file 1: Supplementary Tables S1, S3–5.** DNA samples analysed and variants identified by WGS; TP53 variants identified by TAm-Seq and distribution of variants by clinical outcome.

**Additional file 2: Supplementary Table S2.** Genomic variants targeted for deep sequencing.

**Additional file 3: Supplementary Figure S1-S3.** Probe design strategy for panel sequencing and primer/amplicon map for TAm-Seq; clinical outcomes of additional patients.

**Additional file 4: Supplementary Methods.** Detailed description of TAm-Seq methodology.

## Data Availability

Data are available upon request. Bam files for WGS, targeted panel sequencing and TAm-Seq will also be available through EGA, and further details can be obtained by contacting the corresponding author.

## Abbreviations

ADT: Androgen deprivation therapy
BFS: Biochemical recurrence-free survival
CI: Confidence interval
CNA: Copy number aberration
mCRPC: Metastatic castration-resistant prostate cancer
CTC: Circulating tumour cell
ctDNA: Circulating tumour DNA
HR: Hazard ratio
IRB: Institutional Review Board
ISUP: International Society of Urological Pathology
MFS: Metastasis-free survival
MRI: Magnetic resonance imaging
PSA: Prostate-specific antigen
PSMA-PET: Prostate-specific membrane antigen-positron emission tomography
RARP: Robot-assisted radical prostatectomy
SNV: Single nucleotide variant
SV: Structural variant
TAm-Seq: Tagged-amplicon deep sequencing
UMI: Unique molecular identifier
WGS: Whole-genome sequencing
