# Supplementary material for: Detection of ctDNA in plasma of patients with clinically localised prostate cancer is associated with rapid disease progression
Source: Genome Med. 2020 Aug 17;12:72. doi: 10.1186/s13073-020-00770-1 (PMC7430029; doi:10.1186/s13073-020-00770-1)
Supplement: Supplementary file 1 — Additional file 1: Supplementary Tables S1, S3–5. DNA samples analysed and variants identified by WGS; TP53 variants identified by TAm-Seq and distribution of variants by clinical outcome. [file 13073_2020_770_MOESM1_ESM.docx]

**Supplementary Tables**

**Table S1:** Breakdown of DNA samples analysed per patient

| **Patient** | **Tumour DNA** | **Germline DNA** | **ctDNA a** | **ctDNA b** | **ctDNA c** |
| --- | --- | --- | --- | --- | --- |
| 11193_2 | y | y | y | y | n |
| 11196_3 | y | y | y | y | n |
| 11199_5 | y | y | y | y | y |
| 11201_4 | y | y | y | y | y |
| 11219_6 | y | y | y | y | y |
| 7 | n | n | y | y | y |
| 8 | y | n | y | y | y |
| 11231_9 | y | y | n | n | y |
| 11242_10 | y | y | y | n | y |
| 11243_11 | y | y | y | y | y |

|  | Sample processed – all ctDNA time points available |
| --- | --- |
|  | Sample processed – some ctDNA time points available |
|  | Sample not processed – samples for WGS not available |
| y | Sample available |
| n | Sample not available |

**Table S2.** List of genomic variants targeted for deep sequencing by patient (see attached excel file).

**Table S3**. Summary of tumour related genomic changes identified by whole genome sequencing.

| **Sample** | **Est. Purity %** | **Est. Chr. Count** | **SNVs** | **Indels** | **CNAs** | **SVs** |
| --- | --- | --- | --- | --- | --- | --- |
| 11193_2 | 63 | 44.02 | 14228 | 4238 | 63 | 143 |
| 11196_3 | 67 | 41.32 | 13059 | 4639 | 74 | 209 |
| 11199_5 | 71 | 43.67 | 12112 | 2729 | 56 | 236 |
| 11201_4 | 35 | 44.18 | 17426 | 2045 | 36 | 130 |
| 11219_6 | 42 | 43.24 | 12026 | 3144 | 22 | 80 |
| 11231_9 | 22 | 44.44 | 10225 | 2107 | 0 | 51 |
| 11242_10 | 31 | 43.59 | 11652 | 2704 | 8 | 63 |
| 11243_11 | 55 | 44.12 | 38243 | 5493 | 43 | 105 |

**Table S4.** Summary of TP53 variants showing the length of the amplicon within which they were detected and their predicted pathogenicity.

| **chrom** | **pos** | **ref** | **alt** | **amplicon_length** | **Predicted effect** |
| --- | --- | --- | --- | --- | --- |
| chr17 | 7578475 | G | A | 201 | Pathogenic |
| chr17 | 7574032 | A | T | 195 | Likely pathogenic |
| chr17 | 7578218 | T | C | 194 | Likely pathogenic |
| chr17 | 7577121 | G | A | 190 | Pathogenic |
| chr17 | 7577599 | C | A | 185 | Likely pathogenic |
| chr17 | 7578235 | T | C | 194 | Likely pathogenic |
| chr17 | 7578371 | C | A | 201 | Likely pathogenic |
| chr17 | 7578376 | C | A | 201 | Uncertain |
| chr17 | 7578408 | C | A | 201 | Likely pathogenic |
| chr17 | 7578411 | C | A | 201 | Uncertain |
| chr17 | 7578419 | C | A | 201 | Pathogenic |
| chr17 | 7578420 | C | A | 201 | Likely benign |
| chr17 | 7578423 | C | A | 201 | Likely pathogenic |
| chr17 | 7578449 | C | A | 201 | Likely pathogenic |
| chr17 | 7578463 | C | A | 201 | Likely pathogenic |
| chr17 | 7578492 | C | A | 201 | Likely pathogenic |
| chr17 | 7578513 | C | A | 201 | Likely pathogenic |
| chr17 | 7579418 | G | T | 194 | Uncertain |
| chr17 | 7579483 | C | A | 190 | Uncertain |
| chr17 | 7579529 | C | A | 190 | Uncertain |
| chr17 | 7579586 | G | T | 148 | Uncertain |

**Table S5:** Simple contingency analysis of the frequency of TP53 positive mutations in patients with either biochemical recurrence (BCR) or metastasis.

|  |  | BCR | |  |
| --- | --- | --- | --- | --- |
|  |  | Negative | Positive | Total |
| TP53 variant | Absent | 83 | 84 | 167 |
|  | Present | 7 | 15 | 22 |
|  | Total | 90 | 99 | 189 |

Pearson Chi-square 2.492, p=0.114

|  |  | Metastasis | |  |
| --- | --- | --- | --- | --- |
|  |  | Negative | Positive | Total |
| TP53 variant | Absent | 128 | 39 | 167 |
|  | Present | 12 | 10 | 22 |
|  | Total | 140 | 49 | 189 |

Pearson Chi-square 4.944, p=0.026
