# Supplementary material for: Detection of ctDNA in plasma of patients with clinically localised prostate cancer is associated with rapid disease progression
Source: Genome Med. 2020 Aug 17;12:72. doi: 10.1186/s13073-020-00770-1 (PMC7430029; doi:10.1186/s13073-020-00770-1)
Supplement: Supplementary file 3 — Additional file 3: Supplementary Figure S1-S3. Probe design strategy for panel sequencing and primer/amplicon map for TAm-Seq; clinical outcomes of additional patients. [file 13073_2020_770_MOESM3_ESM.docx]

**Supplementary Figures**

**Figure S1**. Probe design strategy

| - SNVs   - Single probe, covers position of variant - Small Indels   - Single probe, adjacent to event - SVs   - 2 probes, flanking either side of SV | 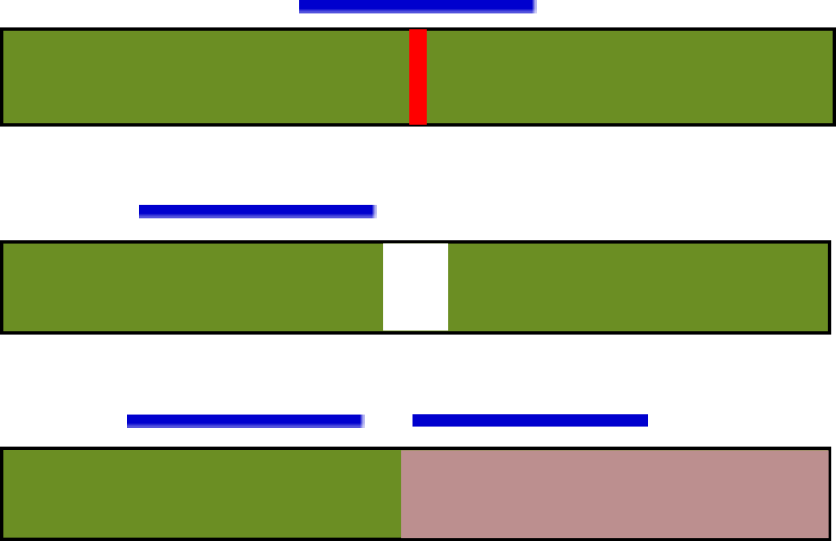 |
| --- | --- |

**Figure S2**. Primers and amplicon mapping used in TAm-Seq analysis

**Figure S3.** Summary of clinical histories of indicated patients post surgery.

**
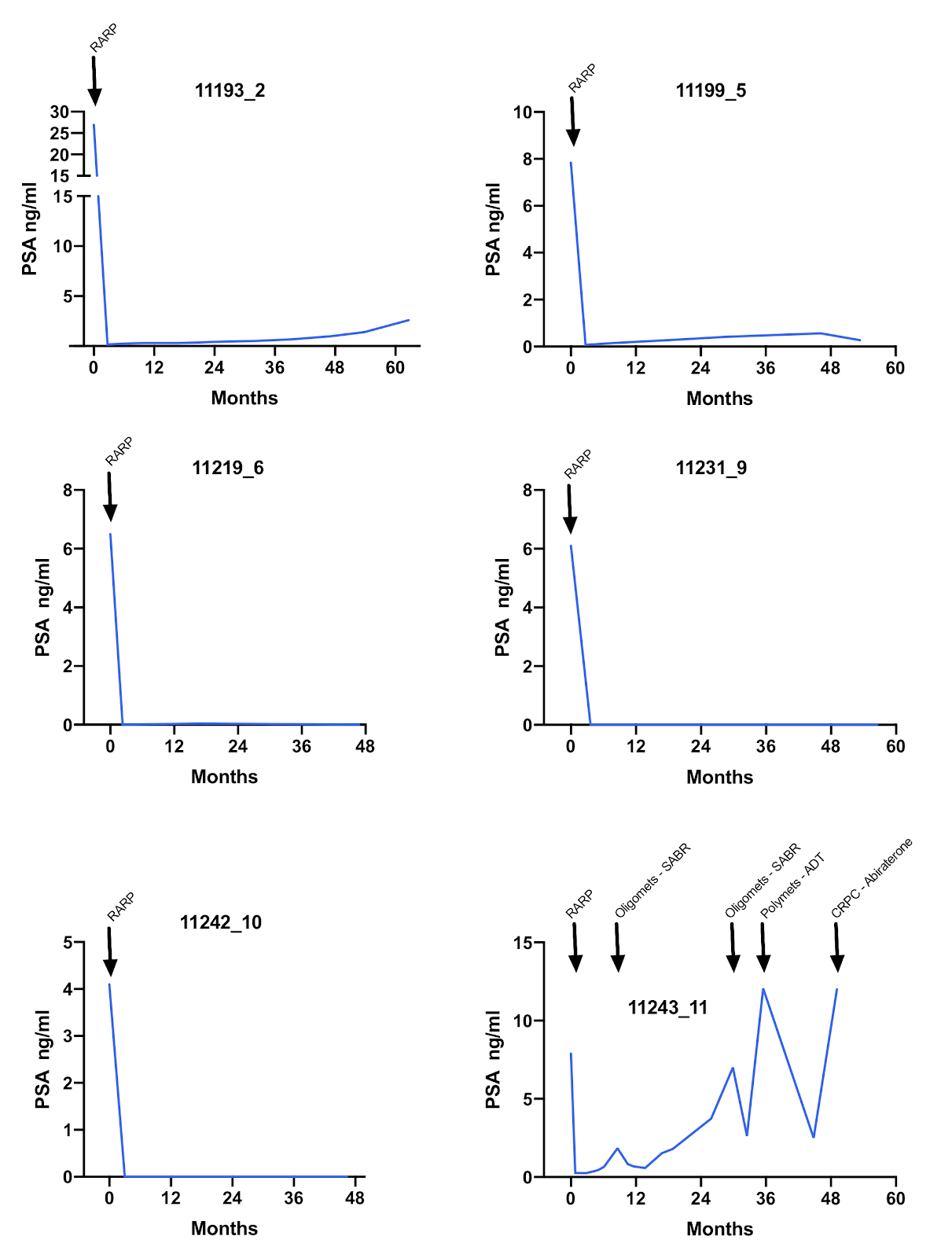
**
