# Supplementary material for: Detection of ctDNA in plasma of patients with clinically localised prostate cancer is associated with rapid disease progression
Source: Genome Med. 2020 Aug 17;12:72. doi: 10.1186/s13073-020-00770-1 (PMC7430029; doi:10.1186/s13073-020-00770-1)
Supplement: Supplementary file 4 — Additional file 4: Supplementary Methods. Detailed description of TAm-Seq methodology. [file 13073_2020_770_MOESM4_ESM.docx]

**TAMSEQ analysis of TP53 mutations in prostate cancer patient plasma.**

Note: Low bind DNA tubes used wherever possible throughout.

**Method 1: Isolation of nucleic acid from patient plasma.**

Overview: Method basically follows the Qiagen QIAamp Circulating Nucleic Acid kit with modifications for economy. Nucleic acid is then quantitated using Qubit.

DNA purification method (cut and pasted from Qiagen QIAamp Circulating Nucleic Acid kit manual with added modifications):

Note: Yield is much better from samples that have been frozen and then thawed than fresh samples.

The volumes given in this protocol suit 1ml plasma as starting material – for different amounts of plasma use the volumes of buffer given in the following table. Wash and elute volumes remain as per 1ml starting volume.

1. Pipet 100 μl, QIAGEN Proteinase K into a 15 ml falcon centrifuge tube.
2. Add 1 ml, of plasma to a 15 ml falcon tube.
3. Add 0.8 ml, Buffer ACL (containing 1.0 μg carrier RNA – I didn’t add this). Close the cap and mix by pulse-vortexing for 30 s. Make sure that a visible vortex forms in the tube. In order to ensure efficient lysis, it is essential that the sample and Buffer ACL are mixed thoroughly to yield a homogeneous solution.

Note: Do not interrupt the procedure at this time. Proceed immediately to step 4 to start the lysis incubation.

1. Incubate at 60°C for 30 min.
2. Place the tube back on the lab bench and unscrew the cap.
3. Add 1.8 ml, Buffer ACB to the lysate in the tube. Close the cap and mix thoroughly by pulse-vortexing for 15–30 s.
4. Incubate the lysate–Buffer ACB mixture in the tube for 5 min on ice.
5. Put VacConnectors into vacuum manifold valves. Insert the QIAamp Mini column into the VacConnector. Insert a 20 ml tube extender (or 20ml Luer Lock syringe barrel – with plunger removed) into the open QIAamp Mini column. Make sure that the tube extender is firmly inserted into the QIAamp Mini column in order to avoid sample leakage.

Note: Keep the collection tube for the dry spin in step 13.

1. Carefully apply the lysate–Buffer ACB mixture from step 7 into the tube extender of the QIAamp Mini column. Switch on the vacuum pump. When all lysates have been drawn through the columns completely, close valve at that connects column to vacuum manifold (or switch off the vacuum pump and release the pressure to 0 mbar). Carefully remove and discard the tube extender.

Please note that sample lysate (4ml) may need up to 5 minutes to pass through the QIAamp Mini membrane by vacuum force.

Note: To avoid cross-contamination, be careful not to move the tube extenders over neighboring QIAamp Mini Columns.

1. Apply 600 μl Buffer ACW1 (or AW1) to the QIAamp Mini column. Leave the lid of the column open, and switch on the vacuum pump. After all of Buffer ACW1 has been drawn through the QIAamp Mini column, close valve at that connects column to vacuum manifold (or switch off the vacuum pump and release the pressure to 0 mbar).
2. Apply 750 μl Buffer ACW2 (or AW2) to the QIAamp Mini column. Leave the lid of the column open, and switch on the vacuum pump. After all of Buffer ACW2 has been drawn through the QIAamp Mini column, close valve at that connects column to vacuum manifold (or switch off the vacuum pump and release the pressure to 0 mbar).
3. Qiagen step not done (Ethanol wash).
4. Close the lid of the QIAamp Mini column. Remove it from the vacuum manifold, and discard the VacConnector. Place the QIAamp Mini column in a clean 2 ml collection tube, and centrifuge at full speed (20,000 x g; 14,000 rpm) for 3 min.
5. Place the QIAamp Mini Column into a new 2 ml collection tube. Open the lid, and incubate the assembly at 56°C for no more than 3 min to dry the membrane completely.
6. Place the QIAamp Mini column in a clean 1.5 ml elution tube (provided) and discard the 2 ml collection tube from step 14. Carefully apply 110 μl of Buffer AVE (or EB) to the centre of the QIAamp Mini membrane. For buffer AVE – ensure buffer is at room temperature, close the lid and incubate at room temperature for 3 min. For buffer EB – pre-warm buffer to 56°C apply 110 μl to membrane and incubate at 56°C for 1min. Spin immediately.
7. Centrifuge in a microcentrifuge at full speed (20,000 x g; 14,000 rpm) for 1 min to elute the nucleic acids.

DNA quantitation using Qubit dsDNA high sensitivity (HS) assay kit: DNA quantitation according to Qubit Molecular Probes dsDNA high sensitivity (HS) assay kit (500 assays Cat#Q32854).

Note: Use only thin wall, clear 0.5ml PCR tubes for assay. Equilibrate solutions to room temperature before use.

1. Make sufficient working solution for samples and two standards of 200. Dilute the dsDNA HS reagent 1:200 in Qubit dsDNA HS buffer
2. For standards add 190 μl working solution and 10 μl standard 1 and 2 to tube and mix well by vortexing but try to avoid bubbles.
3. For samples add 190 μl working solution and 10 μl of sample to give a final volume of 200 μl. Mix well by vortexing.
4. Incubate tubes at room temp for 2 min.
5. Turn on Qubit fluorometer and select dsDNA high sensitivity assay.
6. Select new calibration.
7. Insert tube containing standard 1 into fluorometer, close lid and press “read”. Remove tube and repeat for standard 2.
8. After standards insert samples into fluorometer and “read”.
9. The readout is given as DNA ng/ml in the 200 μl final volume that was assayed – to calculate the sample concentration;

Sample concentration (ng/μl) = Qubit value (ng/ml) X (200/sample μl) / 1000

= Qubit value X 0.02

Expected result: Routinely, I have been getting concentrations around 0.14ng/μl or 15ng total DNA in 110 μl.

**Method 2: TP53 amplicon preparation**

Overview: There are 8 amplicon reactions that are generally prepared as duplexes (2 PCR products). These seem to work better when different primers combinations were made fresh on the day from stocks rather than from stocks containing multiple primers. Primers were 5’ end phosphorylated and HPLC purified so amplicons could be ligated to T-overhang adaptors using off the shelf Klenow Fragment (3’->5’ exo-) for A-tailing and T4 DNA ligase. Amplicons are combined to produce two pools of non-overlapping fragments and purified.

Primer details:

Tm, Score and primer dimer Gibbs free energy calculated using free online tool NetPrimer from Premier Biosoft.

Amplicon mapping:

Larger versions of primer details and amplicon mapping provided at end of document.

Multiplexing Method:

For amplicon production, forward and reverse primers were combined as follows for use in 8 separate PCR reactions per patient. 0.5 μl of each primer/reaction (10 μM stock). Single-plex primers diluted 1:1 with MQ water so that the same amount of liquid is used in setting up the PCR reactions.

E2 + MQ E3 + E9

E4b + E8 E4a + E10

E5a + E11 E4c + E7

E6 + MQ E5b + E12

PCR reaction: Quantities given for single reaction (1X) and to make up master mix for each patient (8.5X) for subsequent addition of primers. Final volume 20 μl.

1X 8.5X

DNA 2 17

Primer (0.5 μl ea @ 10 μM) 2 Primers added to mix aliquots (18 μl)

Q5 DNA pol 2X master mix 10 85

MQ water 6 51

PCR program:

Temp Time Cycles

Initial Denaturation 95 1 min 1

Cycle Denaturation 95 10 sec

Annealing 58 1 sec 38

Extension 72 18 sec

Hold 15 Ꚙ

Amplicon Pooling: Amplicons combined into two pools of non-overlapping products so linkers can be ligated to ends and then further amplified to prepare library. This is done in non-overlapping pools so that concatameric fragments are *not* produced. Pool reactions as follows;

Pool 1 Pool 2

E2 E3 + E9

E4b + E8 E4a + E10

E5a + E11 E4c + E7

E6 E5b + E12

20 μl/PCR reaction X 4 amplicon reactions per pool = 80 μl/pool

Amplicon Clean Up: Half of pool (40 μl) is cleaned up using Qiagen QIAquick PCR purification kit – the other half can be frozen. The following method is cut, pasted and abridged from the QIAquick manual.

1. Add 5 volumes of Buffer PB to 1 volume of the PCR sample and mix (in this case 40 μl PCR pool and 200 μl Buffer PB).
2. If pH Indicator I has been added to Buffer PB, check that the colour of the mixture is yellow.

If the color of the mixture is orange or violet, add 10 μl of 3 M sodium acetate, pH 5.0, and mix. The color of the mixture will turn to yellow.

1. Place a QIAquick spin column in a provided 2 ml collection tube.
2. To bind DNA, apply the sample to the QIAquick column and centrifuge for 30–60 s.
3. Discard flow-through. Place the QIAquick column back into the same tube. Collection tubes are re-used to reduce plastic waste.
4. To wash, add 0.75 ml Buffer PE to the QIAquick column and centrifuge for 30–60 s.
5. Discard flow-through and place the QIAquick column back in the same tube. Centrifuge the column for an additional 1 min.

IMPORTANT: Residual ethanol from Buffer PE will not be completely removed unless the flow-through is discarded before this additional centrifugation.

1. Place QIAquick column in a clean 1.5 ml microcentrifuge tube.
2. To elute DNA, add 60 μl MQ water to the centre of the QIAquick membrane, let the column stand for 1 min, and centrifuge the column for 1 min.

IMPORTANT: Ensure that the elution buffer is dispensed directly onto the QIAquick membrane for complete elution of bound DNA.

Elution efficiency is dependent on pH. The maximum elution efficiency is achieved between pH 7.0 and 8.5.When using water, make sure that the pH value is within this range, and store DNA at –20°C as DNA may degrade in the absence of a buffering agent.

Amplicon quantitation: Quantitate DNA on nanodrop – expect 5-15ng/μl. This concentration is not accurately quantitated on the nanodrop but it is sufficient to provide enough information to set up approximately 100ng DNA for the library prep.

**Method 3: Library Preparation**

Overview: This is based on the NEBNext Ultra II DNA library Prep for Illumina (NEB #7645S/L). The first few steps are different because we have generated amplicons using phosphorylated oligos so they don’t need end repair – they just need to be A-tailed with Klenow Fragment (3’->5’ exo-). Other elements of linker ligation can then be performed using individually sourced components. This method ends up being nearly 10X cheaper than the Illumina kit (components also from NEB). Total number of reactions – 2 pools X 300samples = 600.

Illumina kit E7645L $3914/96X600 = $24,462

Klenow Fragment (3’->5’ exo-) M0212L / 1000U@5U/μl / 200μl / $425

$425/200rxnsX600=$1275

T4 DNA ligase M0202L / 100,000U@400,000U/ml / 250μl / $446 Total = $3015

$446/250rxnsX600=$1070.40

Q5 DNA polymerase M0494L 1250rxns (20μl) / $1395

$1395/1250rxnsX600=$669.60

Starting Material – Amplicon DNA

100ng of DNA (do not combine pools) in 53.5 μl made up with MQ water.

Starting Material – Sequencing Platforms and Primer sets

For test runs on MiSeq Nano (1 million clusters) I have used 12 indexed primers and did 2X150bp paired end sequencing. This returned 1.4million reads that passed filter of a possible 2 million reads (1 million clusters X paired end). This amounts to around 100,000 reads per sample (1.4 million / 12 samples). Evenly distributed across 14 amplicons this should give around 7,000 reads per amplicon (100,000reads / 14amplicons). This held up in practice and was about right (in the original TAMSEQ paper - Forshew et al., 2012 Sci Transl Med 4:136ra36 – each amplicon was theoretically sequenced 3250 times).

For actual runs on MiSeq using reagent kit 2 (15 million clusters) this should produce around 15 times the output of the test run on MiSeq nano. In this test run there were 12 samples so multiplied by 15 = 180 samples. In effect, this will give around twice the number of reads per sample.

1. End Prep

1.1 Add the following components to a sterile nuclease free tube:

100ng Purified amplicon pool DNA: 53.5 μl

NEBuffer 2 (10X): 5 μl

dATP (10 mM): 0.5 μl (0.1 mM final)

Klenow Fragment (3´→ 5´ exo–): 1 μl

Total volume: 60 μl

1.2. Set a 100 μl or 200 μl pipette to 50 μl and then pipette the entire volume up and down at least 10 times to mix thoroughly. Perform a quick spin to collect all liquid from the sides of the tube.

Note: It is important to mix well. The presence of a small amount of bubbles will not interfere with performance.

1.3. Place in a thermocycler, with the heated lid set to ≥ 75°C, and run the following program:

30 minutes @ 37°C

30 minutes @ 65°C

Hold at 4°C

If necessary, samples can be stored at –20°C; however, a slight loss in yield (~20%) may be observed. We recommend continuing with adaptor ligation before stopping.

2. Adaptor Ligation

2.1. Dilute NEBNext Adaptor for Illumina 1:10 (Protocol recommends this be done in 10 mM Tris-HCl, pH 8.0 with 10 mM NaCl but I make up as required in MQ water)

2.2A. Make up Ligation master mix. This dilutes 5X ligation buffer to 1.7X and adds ligase so it is only necessary to pipette one solution rather than two, is easier to manipulate because it is less viscous and gives a volume of 30 μl per reaction as per the NEB protocol. Dilute buffer first and mix well before adding ligase then mix again. The following is to make 30 μl which is sufficient for one reaction.

5X ligation buffer (recipe follows protocol) 10 μl

MQ water 19 μl

T4 DNA ligase 1 μl

2.2B. Add the following components directly to the End Prep Reaction Mixture:

End Prep Reaction Mixture (step 1.3 in section 1) 60 μl

Ligation master mix (step 2.2a above) 30 μl

NEBNext Adaptor for Illumina (dilute 1:10) 2.5 μl

2.3. Set a 100 μl or 200 μl pipette to 80 μl and then pipette the entire volume up and down at least 10 times to mix thoroughly. Perform a quick spin to collect all liquid from the sides of the tube. (Caution: The NEBNext Ultra II Ligation Master Mix is very viscous. Care should be taken to ensure adequate mixing of the ligation reaction, as incomplete mixing will result in reduced ligation efficiency. The presence of a small amount of bubbles will not interfere with performance).

2.4. Incubate at 20°C for 15 minutes in a thermocycler with the heated lid off.

2.5. Add 3 μl of • (red) USER™ Enzyme to the ligation mixture from Step 2.3.

2.6. Mix well and incubate at 37°C for 15 minutes with the heated lid set to ≥ 47°C.

**SAFE STOP** Samples can be stored overnight at –20°C.

3.1 Size selection or clean-up of adaptor ligated DNA

This method is taken directly from the NEB manual and is optimised to return fragments with a 200bp insert.

3.1.1. Vortex SPRIselect beads to resuspend. AMPure XP beads can be used as well. If using AMPure XP beads, please allow the beads to warm to room temperature for at least 30 minutes before use.

3.1.2. Add 40 μl (~ 0.4X) of resuspended SPRIselect beads to the 96.5 μl ligation reaction. Mix well by pipetting up and down at least 10 times. Be careful to expel all of the liquid out of the tip during the last mix. Vortexing for 3-5 seconds on high can also be used. If centrifuging samples after mixing, be sure to stop the centrifugation before the beads start to settle out.8

3.1.3. Incubate samples on bench top for at least 5 minutes at room temperature.

3.1.4. Place the tube/plate on an appropriate magnetic stand to separate the beads from the supernatant. If necessary, quickly spin the sample to collect the liquid from the sides of the tube or plate wells before placing on the magnetic stand.

3.1.5. After 5 minutes (or when the solution is clear), carefully transfer the supernatant containing your DNA to a new tube (Caution: do not discard the supernatant). Discard the beads that contain the unwanted large fragments.

3.1.6. Add 20 μl (0.2X) resuspended SPRIselect beads to the supernatant and mix at least 10 times. Be careful to expel all of the liquid from the tip during the last mix. Then incubate samples on the bench top for at least 5 minutes at room temperature.

3.1.7. Place the tube/plate on an appropriate magnetic stand to separate the beads from the supernatant. If necessary, quickly spin the sample to collect the liquid from the sides of the tube or plate wells before placing on the magnetic stand.

3.1.8. After 5 minutes (or when the solution is clear), carefully remove and discard the supernatant that contains unwanted DNA. Be careful not to disturb the beads that contain the desired DNA targets (Caution: do not discard beads).

3.1.9. Add 200 μl of 80% freshly prepared ethanol to the tube/plate while in the magnetic stand. Incubate at room temperature for 30 seconds, and then carefully remove and discard the supernatant. Be careful not to disturb the beads that contain DNA targets.

3.1.10. Repeat Step 3.1.9 once. Be sure to remove all visible liquid after the second wash. If necessary, briefly spin the tube/plate, place back on the magnet and remove traces of ethanol with a p10 pipette tip.

3.1.11. Air dry the beads for up to 5 minutes while the tube/plate is on the magnetic stand with the lid open.

Caution: Do not overdry the beads. This may result in lower recovery of DNA target. Elute the samples when the beads are still dark brown and glossy looking, but when all visible liquid has evaporated. When the beads turn lighter brown and start to crack they are too dry.

3.1.12. Remove the tube/plate from the magnetic stand. Elute the DNA target from the beads into 22 μl of 10 mM Tris-HCl or 0.1X TE.

3.1.13. Mix well on a vortex mixer or by pipetting up and down 10 times. Incubate for at least 2 minutes at room temperature. If necessary, quickly spin the sample to collect the liquid from the sides of the tube or plate wells before placing back on the magnetic stand.9

3.1.14. Place the tube/plate on a magnetic stand. After 5 minutes (or when the solution is clear), transfer 20 μl to a new PCR tube for (amplification).

**SAFE STOP** Samples can be stored at –20°C.

4.1 PCR amplification

4.1.1. Add the following components to a sterile strip tube:

4.1.1A Forward and reverse primer not already combined (for test runs)

Adaptor ligated DNA fragments from 3.1.14 20 μl

Q5 2X master mix 25 μl

Index Primer (10 μM) 2.5 μl

Universal PCR Primer (10 μM) 2.5 μl

Total volume 50 μl

4.1.1B Forward and reverse primer already combined (for 96 indexed primers)

Adaptor ligated DNA fragments from 3.1.14 20 μl

Q5 2X master mix 25 μl

Index/Universal Primer combined (10 μM) 5 μl

Total volume 50 μl

4.1.2. Set a 100 μl or 200 μl pipette to 40 μl and then pipette the entire volume up and down at least 10 times to mix thoroughly. Perform a quick spin to collect all liquid from the sides of the tube.13

4.1.3. Place the tube on a thermocycler and perform PCR amplification using the following PCR cycling conditions:

Temp Time Cycles

Initial Denaturation 98 30 sec 1

Cycle Denaturation 98 10 sec

8 cycles

Annealing/Extension 65 75 sec

Final Extension 65 5 min

Hold 4 Ꚙ

4.1.4. Proceed to clean-up of PCR Amplification in Section 5.

5. Clean-up of PCR reaction

5.1. Vortex SPRIselect beads to resuspend. AMPure XP beads can be used as well. If using AMPure XP beads, allow the beads to warm to room temperature for at least 30 minutes before use.

5.2. Add 45 μl (0.9X) resuspended SPRIselect beads to the PCR reaction. Mix well by pipetting up and down at least 10 times. Be careful to expel all of the liquid out of the tip during the last mix. Vortexing for 3-5 seconds on high can also be used. If centrifuging samples after mixing, be sure to stop the centrifugation before the beads start to settle out.

5.3. Incubate samples on bench top for at least 5 minutes at room temperature.

5.4. Place the tube/plate on an appropriate magnetic stand to separate the beads from the supernatant. If necessary, quickly spin the sample to collect the liquid from the sides of the tube or plate wells before placing on the magnetic stand.

5.5. After 5 minutes (or when the solution is clear), carefully remove and discard the supernatant. Be careful not to disturb the beads that contain DNA targets (Caution: do not discard the beads).

5.6. Add 200 μl of 80% freshly prepared ethanol to the tube/ plate while in the magnetic stand. Incubate at room temperature for 30 seconds, and then carefully remove and discard the supernatant. Be careful not to disturb the beads that contain DNA targets.

5.7. Repeat Step 5.6 once for a total of two washes. Be sure to remove all visible liquid after the second wash. If necessary, briefly spin the tube/plate, place back on the magnet and remove traces of ethanol with a p10 pipette tip.

5.8. Air dry the beads for up to 5 minutes while the tube/plate is on the magnetic stand with the lid open.

Caution: Do not over-dry the beads. This may result in lower recovery of DNA target. Elute the samples when the beads are still dark brown and glossy looking, but when all visible liquid has evaporated. When the beads turn lighter brown and start to crack they are too dry.

5.9. Remove the tube/plate from the magnetic stand. Elute the DNA target from the beads by adding 33 μl of 0.1X TE.

5.10. Mix well by pipetting up and down 10 times, or on a vortex mixer. In­cubate for at least 2 minutes at room temperature. If necessary, quickly spin the sample to collect the liquid from the sides of the tube or plate wells before placing back on the magnetic stand.

5.11. Place the tube/plate on the magnetic stand. After 5 minutes (or when the solution is clear), transfer 30 μl to a new PCR tube for and store at –20°C.

5.12. Check the size distribution on an Agilent Bioanalyzer High Sensitivity DNA chip. The sample may need to be diluted before loading.

**SAFE STOP** Samples can be stored at –20°C.

6. Library quantitation and combination of pools

6.1 Quantitate library pools on nanodrop. Expect 30-50ng/ μl.

6.2 At this stage pools 1 and 2 for each sample can be combined (providing they are approximately the same concentration).

6.3. This material can be submitted for sequencing. At MHTP each sample will be subject to QC on bioanalyser and combined in equimolar ratio ready for loading onto sequencing flow cells.

**5X Ligation Buffer** [5x] [1X] Stock For 1ml

TRIS pH7.4 250mM 50mM 1M 250μl

MgCl2 50mM 10mM 1M 50μl

PEG (6000-8000) 25% 5% 50% 500μl

rATP (SIGMA cat#A-7699) 5mM 1mM 0.1M 50μl

DTT 5mM 1mM 1M 5μl

MQ 145μl

**Product Details – quick reference guide (prices as per 16/10/2017)**

Eppendorf DNA Lobind tube 1.5ml / SIGMA Z666548-250EA / $16.50

QIAamp Circulating Nucleic Acid Kit (50) / QIAGEN Cat#551144 / 50 rxns / $1839

QIAamp DNA Blood Mini Kit (250) / QIAGEN Cat#51106 / 250rxns / $1451

QIAquick PCR purification kit (250) / Qiagen Cat#28106 / $917

Qiagen Proteinase K (10ml) / Qiagen Cat#19133 / 100rxns / $618

VacConnectors (500) / QIAGEN Cat#19407/ pkt 500 / $261

USER enzyme NEB M5505L 250U@1U/μl / 250μl / $495

NEBNext® Multiplex Oligos for Illumina® (Index Primers Set 1) NEB E7335S / 24 rxns / $183

NEBNext® Multiplex Oligos for Illumina® (96 Index Primers) NEB E6609S / 96 rxns / $1133

NEBNext® Multiplex Oligos for Illumina® (96 Index Primers) NEB E6609S / 384 rxns /$4533

Klenow Fragment (3’->5’ exo-) NEB M0212L / 1000U@5U/μl / 200μl / $425

T4 DNA ligase NEB M0202L / 100,000U@400,000U/ml / 250μl / $446

Q5 DNA polymerase NEB M0494L 1250rxns (20μl) / $1395

Molecular Probes Qubit dsDNA high sensitivity (HS) assay kit / Cat#Q32854 / 500 assays / $466

Agencourt AMPure XP beads Beckman Coulter / A63881 / 60ml / $1681.47

Primer details:

Amplicon mapping:
